# The Determinants of Health-Related Quality of Life in a Sample of Primary School Children: A Cross-Sectional Analysis

**DOI:** 10.3390/ijerph18063251

**Published:** 2021-03-21

**Authors:** Alice Masini, Davide Gori, Sofia Marini, Marcello Lanari, Susan Scrimaglia, Francesco Esposito, Francesco Campa, Alessia Grigoletto, Andrea Ceciliani, Stefania Toselli, Laura Dallolio

**Affiliations:** 1 Department of Biomedical and Neuromotor Science, University of Bologna, 40126 Bologna, Italy; alice.masini7@unibo.it (A.M.); davide.gori4@unibo.it (D.G.); susan.scrimaglia@studio.unibo.it (S.S.); francesco.esposito32@studio.unibo.it (F.E.); alessia.grigoletto2@unibo.it (A.G.); stefania.toselli@unibo.it (S.T.); laura.dallolio@unibo.it (L.D.); 2 Department for Life Quality Studies, University of Bologna, Campus of Rimini, 47921 Rimini, Italy; francesco.campa3@unibo.it (F.C.); andrea.ceciliani@unibo.it (A.C.); 3 Pediatric Emergency Unit, S. Orsola University Hospital, Scientific Institute for Research and Healthcare (IRCCS) Azienda Ospedaliero-Universitaria di Bologna, 40138 Bologna, Italy; marcello.lanari@unibo.it

**Keywords:** health-related quality of life, parent’s proxy report, children, physical sctivity, PedsQl questionnaire, BMI, Actigraph, body image

## Abstract

Background: Health-related quality of life (HRQoL) in childhood is a multidimensional construct with many sub dimensions of subjective experience, including physical activity (PA), psychological well-being, social interaction, and school performance, that represents a fundamental health outcome to assess a child’s physical and psycho-social functioning. Our study aims to explore the potential predictors of children’s health-related quality of life, using a convenience sample from the Imola Active Break Study (I-MOVE), considering demographic, anthropometric measures, PA level measured by Actigraph accelerometers, parent-reported/self-reported HRQoL, and body image. Methods: A cross-sectional analysis was conducted among 151 primary school children in Italy. HRQoL was assessed using the Italian version 4.0 of the Paediatric Quality of Life (PedsQL) questionnaire. Results: Children who spent more time partaking in moderate PA were associated with a higher total PedsQL score (*p* < 0.03). Mother’s body mass index (BMI) was the only variable statistically significant associated with the physical health domain of PedsQL. Parent’s proxy-report perception concerning children’s psychosocial health was statistically relevant. The children’s gender, age, and BMI had no association with any of the HRQoL outcomes. Discussion: Parent proxy-report psychosocial health and mother’s BMI should be considered as predictors of HRQoL for the psychosocial and physical domain. PA should be implemented in order to improve the HRQoL of primary school children.

## 1. Introduction

Interest in health-related quality of life (HRQoL) has been growing, especially as z health outcome among children and adolescents, in order to assess their physical and social functioning, mental health, and well-being [1]. Measures of health-related quality of life (HRQoL) assess important aspects of health that are not detected by traditional physiological and clinical measurements. The World Health Organization (WHO) defines quality of life as ‘‘the individual’s perception of their position in life in the context of the culture and value systems in which they live and in relation to their goals, expectations, standards and concerns”, in other words, a global view that considers many dimensions of human beings [2]. Health-related quality of life (HRQoL) is a multidimensional construct with many sub dimensions of subjective experience, including physical activity, psychological well-being, social interaction, and school performance. In summary, the HRQoL reflects a personal self-evaluation and perception of well-being, enjoyment, and satisfaction with life, general health and functioning [3,4].

Many factors are associated with HRQoL in children, such as demographic (i.e., age and gender) and socio-economic characteristics of the family environment (i.e., parental education level, working situation’s parents, family wealth, housing status, and urbanization rate). Williams et al. described that children of mothers with a low educational level had lower HRQoL scores associated with higher weight categories than children with mothers of a higher education [5]; Costa et al. confirmed that father’s and mother’s educational level and current work status is associated with lower mean scores for all dimensions of HRQoL [6].

Regarding age and gender, Keating et al. showed that adolescents report a lower HRQoL than children, and girls have a lower HRQoL than boys [7].

Wu et al. suggested that elevated levels of physical activity are associated with higher HRQoL, whereas a low level of PA and higher time spent in sedentary behavior are inversely related to health-related quality of life among children and adolescents [8]. Given that HRQoL is multidimensional, it is feasible that some dimensions may be more affected by the weight condition or PA level.

Many well-established studies have reported the benefits of physical activity (PA), including obesity prevention, skeletal health, mental, and emotional and psychological health in children [9,10,11,12,13,14,15]. The World Health Organization recommends children and adolescents aged 5–17 perform at least 60 min of moderate-to-vigorous PA (MVPA) per day [16]. However, this PA guideline recommendation has not been achieved by the majority of young people worldwide [17,18,19,20,21,22,23]. In many European Union countries, the number of inactive children (not compliant to recommendations) is constantly growing, particularly in Italy: it is estimated that only 9.5% of boys and 2.6% of girls reach the daily amount of PA [24]. Children and adolescents spend more time engaging in sedentary activities than a decade ago because of the increasing use of screen-based electronic devices (e.g., smart phones and laptops) and widespread access to the internet [25]; this type of sedentary behavior may have a significant impact on health, as it is independently associated with weight status and obesity [26]. Nowadays, obesity is a public health problem in many countries, not only among adults, but also in children. In Italy, the most recent data from the national surveillance system reported 21.3% of children as overweight and 9.3% of children as obese, including 2.1% of children being severely obese [24,27]. The prevalence of overweight and obesity among children, although there has been a decrease from 2008 to 2016, still remains one of the highest in Europe. In 2015, the United Nations established “The Sustainable Development Goals”, in order to identify the prevention and control of non-communicable diseases as one of the most important priorities. Among the non-communicable disease risk factors, obesity is particularly concerning and has the potential to negatively interfere with many of the health benefits that have contributed to increased life expectancy [28]. Currently, it is widely known that overweight and obesity entail health consequences, not only limited to physical health, but to other problems including body dissatisfaction, negative body image, low self-esteem, depression, stigmatization, and social marginalization, which can influence psychological and social health issues [29]. Body image is a multidimensional construction reflecting a mental representation of one’s own body appearance, not linked to the actual physical appearance, that is likely to affect other domains of psychological health; [30,31] furthermore, body image distortion cannot allow for perceiving the objective size of body [32], and this occurs when the perception of a body or parts of a body do not satisfy the image determined culturally or socially [33].

Obesity-related complications and comorbidity represent major public health concern, even in children, but also have nonmedical short-term consequences such as impacting on psycho-social well-being and quality of life, which may be relevant [34,35]. A recent review suggests that a higher weight status has a moderate to strong negative influence on overall HRQoL in paediatric populations [36]; Tsiros et al. found an inverse linear relationship between HRQoL and body mass index (BMI) for most of their included studies. Other potential determinants of children’s health-related quality of life could be parents’ perspective about their child’s health, because it is likely to be a strong driver. The parent–child agreement regarding health-related quality of life in general is low–moderate and appears to change as the child ages. Clearly, there is an increasing need to take into account parents’ evaluation about their children’s quality of life in order to be able to detect and take action in case of signs of decreased HRQoL in children at an early stage [37].

To the best of our knowledge, few studies have evaluated parents’ and children’s perspective Quality of Life [5] and none have been performed in Italian community samples of primary school children.

Considering the importance of investigating HRQoL in these types of populations, we decided to conduct a cross-sectional study, which forms part of the multiple targeted research protocol for a quasi-experimental trial in primary school children based on an active break intervention—the Imola Active Breaks (I-MOVE) Study [38].

The present cross-sectional study aims to explore anthropometric measures, parent’s education, physical activity level, parent-reported/self-reported HRQoL, and body image as potential predictors of children’s health-related quality of Life, using a convenience sample from the Imola Active Break Study project (I-MOVE). We hypothesized that children’s HRQoL is mostly influenced by objectively measured physical activity and BMI. In particular, we expected that, in our sample, children spending more time engaging in physical activity and/or with a lower BMI would have a better HRQOL.

The innovative component and added value of this study consisted firstly of assessing parents’ HRQoL and the parents’ perspective of their child’s quality of life, as well as exploring body image as potential determinants of children’s HRQoL, and secondly, of analyzing the physical activity levels using objective parameters from accelerometers, which allowed for analyzing not only the amount of 60-min recommended per day, but also the different intensities as well as other measures (moderate, vigorous, light intensity of physical activity, MVPA, sedentary time, and step count). This is an important added value as most of the literature regarding this field assessed the PA level using only self-reported methods [8].

## 2. Materials and Methods

### 2.1. Study Design and Participant

The present study is a cross-sectional analysis employed using baseline data collected from the I-MOVE quasi experimental study [38] conducted between October 2019 and December 2019, set in a primary school of the Emilia Romagna region, Imola (Italy).

The I-MOVE study was endorsed by the University of Bologna (Italy). The University of Bologna Bioethics Committee, on 18 March 2019, approved the study (Prot. n. 0054382 of 18 March 2019—(UOR: SI017107-Classif. III/13)). The study was conducted following the Declaration of Helsinki and was approved by the school board. We used the following inclusion/exclusion criteria: (i) attending from the 1st to 5th grade (aged 6–11 years); (ii) not having health problems or physical disability, which might limit PA performance; and (iii) having obtained informed consent of parents and permission for personal data processing.

We enrolled 151 children aged 6–10. A total of 110 children had all of the variables we assessed. If children were absent or unavailable during the study period, they were classified as “missing” [38].

### 2.2. Study Variables and Instruments

Data were collected using different tools, namely: questionnaires, and anthropometric and physical activity assessments. All of the children and parents who agreed to participate in the study underwent evaluations. The questionnaires could be filled by both parents.

#### 2.2.1. Health-Related Quality of Life

Health-related quality of life was assessed using the Italian version 4.0 of the Pediatric Quality of Life Inventory (PedsQL) [39]. Children self-completed the PedsQL questionnaire in classroom after instructions given by the research team.

We used self-reported children’s HRQoL, self-reported adult HRQoL total score and parents perceived children’s HRQoL total score diversified according to the children’s age (8–12- and 5–7-year-old children) [40,41,42]. Khairy et al. study recommended the use of PedsQL questionnaire as a simple, easy and reliable measurement model for assessment of Health-Related Quality of Life [43].

The PedsQL questionnaire had 23-items (Total PedsQL) divided into 4 domains that were used to assess the children’s level of difficulty in Physical Functioning (PF-8 items) and Psychosocial health divided into Emotional Functioning (EF-5 items), Social Functioning (SF-5 items) and School Functioning (SchF-5 items).

As stated by Varni et al., every item was reversely scored and linearly transformed to a 0–100 scale, so that higher scores indicate better HRQoL (Health-Related Quality of Life) [39]. To reverse score, we transformed the 0–scale items to 0–100 as follows: 0 = 100, 1= 75, 2 = 50, 3 = 25, and 4 = 0.

#### 2.2.2. Demographic Variables

Parents or guardians answered to a series of socio-demographic and anthropometric questions (e.g., gender, education level, weight and height) inside the ZOOM8 validated Questionnaire [44,45].

#### 2.2.3. Body Image Perception

To evaluate the parent’s body image perception about their children, each family was invited to choose among a set of silhouettes which one best identified the body image of their children [46]. Parents selected two different silhouettes: the first regarded the image that they believed was the most similar to their children (‘actual figure’) and the second was the image that parents desired for their children (‘ideal figure’). Mother’s Feel Ideal Difference (FID) index was calculated by subtracting the score of the ideal figure chosen by the parents (mother) from the figure chosen as representative for their child. A positive FID score indicates the actual figure was bigger than the ideal figure and a negative score indicates the actual figure was thinner than the ideal figure. A FID score of 0 indicates no discrepancy (same figure chosen as actual and as ideal).

Improper perception of weight status of their children was evaluated by means of Mother’s FAI (Feel weight status minus Actual weight status Inconsistency): we calculated the inconsistency score FAI by subtracting the conventional code assigned to the actual weight status of the participant (1 = underweight; 2 = normal weight; 3 = overweight; 4 = obese) from the code that her/his mother perceived. A FAI score of zero indicates no inconsistency in weight status perception; a positive score indicates that weight status is overestimated whereas, a negative score indicates that weight status is underestimated.

#### 2.2.4. Anthropometric Variables

Six anthropometric characteristics (height, weight, waist and hip circumferences, triceps and subscapular skinfold thicknesses) were collected according to standardized procedures [47,48]. In particular, height was measured to the nearest 0.1 cm using a portable stadiometer (SECA 217, SECA: Hamburg, Germany). Body weight was measured to the nearest 0.1 kg (light indoor clothing, without shoes) using a calibrated electronic scale (SECA 877: Hamburg, Germany).

Waist circumference (WC) was measured to the nearest 0.1 cm with a non-stretchable tape (GPM measuring tape; DKSH Switzerland Ltd.: Zurich, Switzerland): WC was measured between the lowest rib and the iliac crest.

Waist and height were used to calculate the Waist/height ratio (WtHR) to stratify children in two categories (0.5 and >0.5); the value of 0.5 is chosen as the cut-off of cardiovascular risk [49,50,51]. BMI was calculated as weight (in kilograms) divided by the square of height (in meters). This index was used to assess the weight status of each participant according to Cole cut off values by sex and age [52,53].

Triceps (TSF) and subscapular (SSF) skinfold thickness was measured to the nearest 0.1 cm on the left side with a Lange caliper (Beta Technology Inc.: Santa Cruz, CA, USA). TSF was measured midway between the tip of the acromion and olecranon processes, while SSF raising an oblique skinfold below the inferior angle of the scapula at 45° to the horizontal plane following the natural cleavage lines of the skin. TSF and SSF were evaluated according to the Frinsancho cut off (2008) [54]. Body composition parameters (percentage fat (%F), fat mass (FM) and fat free mass (FFM)) were calculated using the skinfolds equations of Slaughter et al. (1988) [55].

#### 2.2.5. Physical Activity Variables

The time spent in physical activity and sedentary behaviour was monitored through Actigraph accelerometers (Actigraph, LLC, Pensacola, FL, USA) (ActiLife6 wGT3X-BT set to 10-s epochs). The accelerometer data were analysed through ActiLife 6.13.3 software (ActiGraph, LCC, Pensacola, FL, USA), with an epoch length set to 10 s to allow a more detailed estimate of PA intensity [56].

The data processing procedures to evaluate total time spent in sedentary behaviour and PA at different intensities are consistent with previous studies on children and adolescents [17,57,58]. The children wore the accelerometers, around the waist in the right side, with an elastic belt [59], over a seven-day period (five weekdays and two weekend days), and removed them only when bathing, swimming and showering. We computed the accelerometer’s data only when children were complying with some specific inclusion criteria such as: having worn the accelerometer for at least 10 h every day (sleeping hours included) during 3 weekdays and 1 weekend day). The Evenson cut points were used to calculate the minutes spent in physical activity (light, moderate and vigorous) per day [60].

### 2.3. Statistical Analysis

All analyses were carried out using SPSS, version 22 (Statistical Package for Social Science) (SPSS Inc. Chicago, IL, USA) and STATA, version 13 (StataCorp 2013. Stata Statistical Software Release 13. College Station, TX: StataCorp LP). Categorical variables are presented as frequency (percentage) and continuous variables are presented as means and standard deviation (SD).

We applied multiple linear regression to determine the associated factors of health-related quality of life. Using backwards stepwise analysis all variables were tested for use in the final multiple linear regression based not only in univariate analysis but also on principles of parsimony and biological plausibility. The significance level was set to *p* ≤ 0.05.

## 3. Results

### 3.1. Study Participants

Table 1 shows the main participants’ characteristic.

Mean age was 7.77 (SD = 1.42) with 54.2% male. The prevalence of normal-weight and overweight/obese were 67.5% and 32.5% respectively.

The 46.2% of mother and 55.5% of father had a medium education level.

PedsQL Total Score in the sample was 72.02 (SD = 13.284); the average score of Physical Health domain was 74.25 (SD = 13.83), the Psychosocial Health domain was 70.79 (SD = 15.87) the Emotional Functioning was 67.26 (SD = 21.19), the Social Functioning was 74.97 (SD = 18.71) and the School Functioning was 70.18 (SD = 19.09). Using Cole cut-off, the sample was stratified in three categories [52,53]. The 67.5% of the sample was normal weight, 21.9% overweight and 10.6% obese. Stratifying children with WtHR, the 83.9% were categorized as not at risk and 16.9% were at cardiovascular risk. Considering Actigraph parameters, the mean daily MVPA was 54.67 (SD = 21.24) in fact exclusively the 38.8% of children met the recommended level of 60 min of PA per day.

Considering body image, the 57.8% of parents have an adequate body image perception of their child, the 34.4% underestimated while the 7.8% overestimated.

### 3.2. Linear Regression Analysis

A stepwise analysis was performed in order to investigate the variables that were to be used in the final regression represented in the adjusted conditions in Table 2 and Table 3A,B. As is usually done, the variables of age, BMI, and gender were used in all of the regression analyses.

Regarding the total score of the PedsQL, a stepwise analysis revealed that the mother’s BMI, parent’s proxy-reports PedsQL total score, levels of physical activity, and body image were relevant to include in the model. Indeed, adjusted linear regression showed that a moderate PA was statistically significant positively associated with the total PedsQL score (*p* < 0.05). Moreover, concerning sedentary time, the regression analysis results reported a positive significant association (*p* < 0.05) with total PedsQL score (Table 2). The R-square was 0.1421.

In the physical health domain (Table 3A), the regression analysis was adjusted for mother’s BMI, and adult self-reported physical health after performing stepwise in reverse. Mother’s BMI was the only statistically significant variable negatively associated with the physical health domain of the children (*p* = 0.02). The R-square was 0.1945.

Table 3B shows the psychosocial health domain adjusted for body image, parent proxy-reports of psychosocial health, and levels of physical activity showed a significant (*p* < 0.01) positive association between parent’s proxy-report and the children’s psychosocial Health domain. The R-square was 0.1421.

Interestingly, the children’s gender, BMI, and age had no association with any of the HRQoL outcomes.

## 4. Discussion

The aim of this study was to investigate the anthropometric measures, parent’s education, physical activity level, parent-reported/self-reported HRQoL, and body image as potential predictors of children’s health-related quality of life from a sample of primary school children.

The main significant finding of this study was that a higher time spent in moderate intensity physical activity was positively associated with a higher health-related quality of life in children measured by the total PedsQL score. These results were in line with previous studies [8,19,61]. Indeed, the systematic review by Wu et al. of meta-analyses confirmed that children with higher levels of physical activities had a better HRQoL [19]. Furthermore, this PA and HRQoL’s positive association was consistent, regardless of weight status, age, socio-economic characteristics, and sex [8]. Within our sample, weekly moderate physical activity levels, measured by objective tools, were significant predictors of health-related quality of life considering the PedsQL total score.

In addition, Wafa et al. found a positive relationship between HRQoL and MVPA, indicating that children who were physically active had a better quality of life [19]; although in their regressions, after the adjusted analyses, the relationship seemed to not be significantly relevant. Conversely, in our study, the relationship between the total PedsQL score and higher levels of moderate intensity physical activity was still significantly confirmed by the adjusted linear regression.

Contrary to most of the studies focused on quality of life, we found that gender and age were not a significant factor associated with variations in HRQoL. Our results are in accordance with Khairy et al. [43], who found that gender differences in HRQoL were probably not very relevant in childhood, but started to appear or increase in adolescence, especially among girls; in addition, they found that for age, adolescence most likely affects the quality of life rather than primary school age. As suggested by Tsiros et al. [36], it is essential to take into account the parent’s perceived HRQoL for their child. Indeed, in our sample, it was observed that the parent’s proxy-report child’s psychosocial health was significantly positively associated with the child’s self-reported psychosocial health. Williams et al., however, found less agreement between parent proxy and child-reported HRQoL in a 12-year-old age group compared with younger children [5]. Therefore, HRQoL perceptions between children and parents likely begin to differ when their age increases, as the child develops a more complex and independent understanding of the world, rather than accepting their parental opinions [36]. Regarding the physical health domain, we found a statistically significant inverse association between mother’s BMI and child’s physical health. To the best of our knowledge, there are no studies that consider parent’s anthropometric measures as predictive factors of quality of life in the physical health domain. The scientific literature confirms that the parent’s BMI is associated with children’s BMI [62] and health behavior [63]; therefore, considering that the family and environmental contexts affect children’s lifestyle, it was not misleading to believe that the mother’s BMI might be considered as a predictor of the children’s health-related quality of life.

Concerning the weight status scope, the majority of the studies in this field showed that a higher weight status had a moderate to strong negative influence on the overall HRQoL in pediatric populations [3,19,36]. Nevertheless, the children’s sample in our study did not report a significant difference in quality of life related to different BMIs. Similarly, some smaller USA studies did not find a significant relationship between BMI and quality of life [64,65,66]. This controversial result may likely be due to some limitations, such as the small sample size, which presents a bias in favor of normal-weight subjects. Indeed, only a small part of our sample presented with obesity, and this probably influenced our results. A further finding not in line with the literature was the positive association between time spent engaging in sedentary behavior and the total PedsQl score. A possible explanation might be related to the method used to assess the time spent participating in sedentary activities, which was not detected from the child’s daily sleep hours. It is likely that, for this reason, there was no inverse association between time spent in sedentary behavior and children’s health-related quality of life. Nevertheless, the use of accelerometers is the most widely accepted method of objectively measuring time spent engaging in PA in youth [67].

## 5. Conclusions

The findings from this cross-sectional study suggest that parent’s proxy-report of child’s psychosocial health and mother’s BMI are potential predictors of a child’s health-related quality of life for the psychosocial health and physical health domains, respectively.

For these reasons, future research should consider parent’s proxy-report and parent’s anthropometric characteristics as factors potentially associated with HRQoL in children.

Furthermore, in our primary school children sample, moderate physical activity was positively associated with the general PedsQL score, confirming itself as a predictor of HRQoL.

Given this positive association found between children’s moderate weekly PA and their HRQoL, these results indicate that supporting the implementation of PA intervention, such as active commuting to and from school, active school recess, active breaks during the curriculum and extra-curriculum time, and health-related homework programs on physical activity, may provide benefits in terms of children’s quality of life.

Finally, we believe that there is a need to study the short and long-term effects of structured physical activity interventions on health-related quality of life, especially using school settings.

## Figures and Tables

**Table 1:** Baseline characteristics of the participants.

Characteristics	Total Sample (*n* = 151) N (%) or Mean ± DS
General Information	
Age	7.77 ± 1.42
Gender (male)	*n* = 83 (54.2%)
Mother’s education	
*Low*	*n* = 21 (16.2%)
*Medium*	*n* = 60 (46.2%)
*High*	*n* = 49 (37.7%)
Father’s education	
*Low*	*n* = 26 (20.3%)
*Medium*	*n* = 71 (55.5%)
*High*	*n* = 31 (24.2%)
Mother’s BMI	22.68 ± 3.15
Father’s BMI	26.52 ± 3.59
Anthropometric measures	
BMI	17.74 ± 2.72
*Normal-weight*	*n* = 102 (67.5%)
*Over-weight*	*n* = 33 (21.9%)
*Obese*	*n* = 16(10.6%)
%F	16.83 ± 4.98
WtHR	0.46 ± 0.04
*Non at risk*	*n* = 125 (83.9%)
*At risk*	*n* = 24 (16.9%)
Body Image	
Under	*n* = 44 (34.4%)
Correct	*n* = 74 (57.8)
Overestimation	*n* = 8 (7.8%)
Physical Activity Outcome	
Daily MVPA	
*Meet the recommended level*	*n* = 57 (38.3%)
*Not meet the recommended level*	*n* = 92 (61.7%)
Weekly MVPA	328.14 ± 127.22
Weekly Light PA	1661.54 ± 388.77
Weekly Moderate PA	214.31 ± 78.00
Weekly Vigorous PA	113.82 ± 58.33
Weekly Sedentary time	6655.44 ± 489.08
Weekly Total Steps	54,557.73 ± 16,681.01
HRQoL Children	
PedsQl Children Total score	72.02 ± 13.28
PedsQl Physical Health	74.25 ± 13.83
PedsQl Psychosocial Health	70.79 ± 15.87
PedsQl Emotional Functioning	67.26 ± 21.19
PedsQl Social Functioning	74.97 ± 18.71
PedsQl School Functioning	70.18 ± 19.09
HRQoL Parent proxy-reports	
PedsQl Total score	79.19 ± 10.86
PedsQl Physical Health	82.73 ± 13.29
PedsQl Psychosocial Health	77.25 ± 11.87
PedsQl Emotional Functioning	72.96 ± 13.47
PedsQl Social Functioning	81.42 ± 15.15
PedsQl School Functioning	77.29 ± 16.26
HRQoL Adult self-reports	
PedsQl Total score	77.92 ± 9.88
PedsQl Physical Health	77.77 ± 12.55
PedsQl Psychosocial Health	77.97 ± 10.68
PedsQl Emotional Functioning	66.86 ± 15.57
PedsQl Social Functioning	85.71 ± 12.30
PedsQl Work Functioning	81.33 ± 11.45

BMI—body max index; %F—percentage fat mass; WtHR—waist to height ratio; PA—physical activity counts per minute; PedsQl—Pediatric Quality of Life Inventory.

**Table 2:** The associated factors of health-related quality of life in children for the total score domain.

	Total Score (*n* = 109) Reg. Coeff. (95% CI)	*p*-Value *
Gender	−3.26 (−8.46; 1.94)	0.22
Age	0.38(−1.47; 2.4)	0.68
BMI	0.35 (−0.58; 1.28)	0.46
Mother’s BMI	−0.54 (−1.24; 0.15)	0.123
Sedentary ^a^	0.03 (0.00; 0.57)	0.04 *
Light PA ^a^	0.03 (0.00; 0.05)	0.09
Moderate PA ^a^	0.075 (−0.01; 0.14)	0.03 *
Total Parent proxy-reports	0.17 (−0.03; 0.38)	0.10
Body image	3.51 (−0.44; 7.46)	0.08

* Significant *p*-value < 0.05; ^a^ accelerometer measured count per minutes calculated as weekly total minutes spent in sedentary, light, and moderate activity.

**Table 3:** (**A**) The associated factors of health-related quality of life in children for the physical health domain. (**B**) The associated factors of the health-related quality of life in children for the psychosocial health domain.

(A)		
	Physical Health (*n* = 114) Reg. Coeff. (95% CI)	*p*-Value *
Gender	−1.90 (−6.70; 2.89)	0.43
Age	−1.62 (−3.46; 0.22)	0.08
BMI	0.43 (−0.54; 1.39)	0.38
Mother’s BMI	−0.91 (−1.68; −0.15)	0.02 *
Physical health from the adult self-reports	−0.16 (−0.36; 0.03)	0.1
**(B)**		
	** Psychosocial Health (*n* = 115) Reg. Coeff. (95% CI) **	*p*-Value *
Gender	−0.24 (−6.20; 5.72)	0.9
Age	1.87 (−0.27; 4.01)	0.09
BMI	−0.84 (−2.33; 0.66)	0.27
Sedentary ^a^	0.03 (−0.01; 0.06)	0.10
Moderate PA ^a^	0.06 (−0.02; 0.13)	0.14
Light PA ^a^	0.02 (−0.01; 0.06)	0.17
Psychosocial health parent proxy-reports	0.40 (0.18; 0.61)	0.00 *
Body image perception	3.60 (−0.89; 8.18)	0.12

* Significant *p*-value < 0.05; ^a^ accelerometer measured count per minutes, calculated as weekly total minutes spent engaging in sedentary, light, and moderate activity.

## Data Availability

The data presented in this study are available on request from the corresponding author. The data are not publicly available due to ethical and privacy reasons.
